# A fuzzy logic-based risk evaluation and precaution level estimation of explosive, flammable, and toxic chemicals for preventing damages

**DOI:** 10.1016/j.heliyon.2024.e41216

**Published:** 2024-12-16

**Authors:** Abdul Aziz, Md Masum Suzon, Rakib Hasan

**Affiliations:** Department of Computer Science and Engineering, Khulna University of Engineering & Technology, Khulna - 9203, Bangladesh

**Keywords:** Explosiveness, Fuzzy logic, Flammability, Hazardous chemicals, Precaution level, Risk evaluation

## Abstract

Chemical industries are highly vulnerable to accidental events or terrorist attacks due to their processing, storage, and transportation of explosive, flammable, and toxic materials. Major industrial risks include fire, explosion, and toxic chemical release. An effective risk evaluation system is essential to prevent accidents or terrorist attacks. The proposed system can evaluate the risk of handling hazardous chemicals and determine the appropriate precaution level to prevent accidents using a fuzzy inference system. The inventory size, explosiveness, flammability, and toxicity of the chemicals are used as input variables for evaluating the output variable risk. The defuzzified value of risk indicates the amount of damage it can cause if accidents happen. Using the calculated risk value and the location of the chemical industry as input variables, precaution level can be determined. Security arrangements have been recommended based on the different precaution levels so that industries can take appropriate measures according to the calculated results. This approach helps to protect the environment and save lives from any mishaps.

## 1 Introduction

### 1.1 Background

Chemicals are a form of matter with a defined composition and characteristic properties. They play a major part in industries like healthcare, agriculture, textiles, water treatment, clothing, pulp and paper, primary metals, and petroleum refining. The chemical industry has become one of the fastest-growing economies in the world, and as societies advance, the demand for chemicals continues to rise. However, Chemical processes involve the reaction of substances that can pose threats to human health and the environment if they are not stored, utilized, or managed in an appropriate manner. Under certain conditions, such as accidental fires, natural disasters, or lightning strikes, chemicals may pose risks that could result in explosions, fires, or toxic exposure. Chemicals may have acute and chronic impacts on an organism's respiratory, reproductive, cardiovascular, neurological, immunological, and metabolic systems, with effects that may vary in severity and duration based on exposure. Some of the dangerous properties of chemicals are discussed below.

**Explosiveness****[1]****:** An explosive substance is a reactive substance that stores a significant amount of potential energy. An explosion occurs when stored energy abruptly transforms into other forms such as heat, light, sound, and pressure. The pace at which explosive materials dilate can be used to classify them. High explosives are those that blow off at a speed greater than that of sound. On the other hand, whose reaction front moves more slowly than the speed of sound is called low explosive. Explosives can also be categorized based on their sensitivity to heat or pressure. Primary explosives are highly sensitive materials that can be denoted by a small amount of heat or pressure. On the contrary, secondary or tertiary explosives are relatively less sensitive to heat or pressure.

**Flammability****[2]****:** Flammability refers to the ability to ignite in the presence of oxygen, typically requiring an ignite source. The flammability of a chemical is generally determined by its flash point, which is the lowest temperature at which the chemical can emit sufficient vapors to ignite. Chemicals with lower flash points are considered more flammable. Furthermore, the flammable range of a substance defines the concentration limits within which the chemical's vapors, mixed with air, can ignite or explode.

**Toxicity****[3]****:** Toxicity means how harmful a chemical or mix of chemicals can be to a living thing, either to the whole organism or to specific parts like a cell or organ. When a large amount of chemical causes harm, it is considered particularly non-toxic but when a small amount of chemical causes harm, it is considered as highly toxic.

### 1.2 Major industrial risks

The majority of industrial dangers are generally related to a fire spreading out inside the industry or chemicals accidentally spilling out of their containers. Major risks are classified based on the type of chemical emission, root cause of fire and release, and its consequences. Below, are some of the primary industrial risks discussed.

**Fire****[4]****:** The most common risk is fire but it is less severe. Nonetheless, fires can occasionally result in explosions. Combustion processes may release toxic gases, including cyanide, carbon monoxide, and acrolein. Additionally, fires can damage physical structures and critical utilities such as electrical systems and instrumentation, potentially exacerbating the situation. Fire exposure often results in skin burns, which vary in severity depending on the duration and intensity of the heat.

**Explosion****[4]****:** Explosions are often identified from a distance by the audible manifestation of a shock wave. The huge pressure generated by the shock wave has the potential to be fatal. Flying glass, collapsing structures, and debris are major causes of instant casualties and severe injuries. Explosions occur when a large quantity of potential energy is rapidly released, which is generally caused by the quick burning of combustible materials or other chemical reactions that liberate significant heat energy. Such reactions include polymerization, material destabilization, and various exothermic interactions.

**Toxic/Chemical release****[5]****:** Toxic vapors released abruptly into the atmosphere can be extremely dangerous and can cause death or serious injury to people even kilometers away. When these vapors spread into water bodies like rivers and lakes, either directly or through contaminated firefighting water, they can pose a major health risk. When these poisonous compounds are ingested by living beings, they can poison them. When toxic materials enter the body, they can be absorbed into the bloodstream and transported to various organs. In addition to ingestion, toxins can also enter the body through the eyes and skin.

### 1.3 Overview of terrorism and some accidental incidents

Chemical process plants are complex and their different parts depend on each other, it's important to carefully consider the risk factors during risk analysis. Ignoring some of the factors can lead to uncertainties and inaccurate assessments of safety measures. This oversight could increase the chance of major accidents and make their consequences worse.

Moreover, terrorists who have knowledge about the orientation and operations of the industry can create situations that can lead to accidents. This raises a serious security concern about whether current security measures are adequate or need enhancement. Chemical sites situated in densely populated or economically important areas are at risk of potential terrorist attacks. There have been numerous incidents of terrorism and accidents targeting Chemical Process Industries (CPI) and their transportation systems. For instance, a devastating explosion occurred at the Port of Beirut in 2020, resulting in at least 218 fatalities, 7,000 injuries, and approximately US$15 billion in property damage. The blast was caused by around 2,750 tonnes of ammonium nitrate stored in a warehouse without adequate safety measures for six years [6]. Recently in 2022, at least 49 people, including 9 firefighters, lost their lives and over 300 people were injured in a massive fire at a container storage facility in Shitakundo, near the port city of Chattogram in southeast Bangladesh. Firefighters mislabeled the hydrogen peroxide containers, and they extinguished the fires with water rather than foam [7]. There have also been terror attacks related to the chemical industry. For example, in 1997, a group of four Ku Klux Klan members conspired to plant an improvised explosive device on a hydrogen sulfide tank situated at a refinery in Dallas, USA. The intention was to use the explosion as a diversion for their plan to carry out an armored car robbery on the opposite side of the town [8].

The objective of this study is to consider the identified risk factors to assess the risk level and necessary precautions associated with hazardous chemicals, improving on existing evaluation methods. Thus, ensuring adequate safety measures can help prevent accidents and attacks, ultimately protecting lives.

### 1.4 Our contribution

The most important contributions of this research are integrated as follows:

*i.* The vulnerability and quantity of hazardous chemicals are key factors used to evaluate risk in this study using fuzzy logic.
*ii.* The study primarily focuses on the volume and hazardous nature of the chemicals stored or processed.
*iii.* The evaluated risk is combined with the industry location to determine the appropriate precaution level.
*iv.* Specific recommendations are provided based on this precaution level to enhance safety and mitigate potential risks.

## 2 Literature review

The process of risk assessment presents a formidable challenge, requiring consideration of numerous variables that are often difficult to quantify due to varying levels of transparency, accuracy, and precision in available data. Numerous studies have been conducted to develop both quantitative and qualitative techniques for identifying and evaluating the risks associated with explosive and chemical substances. However, the integration of fuzzy logic into risk assessment has demonstrated particular effectiveness. Fuzzy methods are user-friendly and efficient, streamlining the assessment process and saving time. Additionally, fuzzy models are reliable and generate precise and comprehensive results, which can be valuable for future research endeavors. Fuzzy logic provides a natural approach to address these uncertainties while preserving human inventiveness and intuition, which are crucial for effective risk analysis. Fuzzy logic, initially developed by Lotfi A. Zadeh [9], extends traditional set theory and has been widely applied across domains such as control theory, artificial intelligence, and security and safety decision-making [10], [11], [12], [13].

Security risk assessment methodologies often utilize tools like the Security Risk Factor Table (SRFT), which evaluates risks by considering factors such as location, visibility, and inventory [14]. Recent enhancements to the SRFT framework have incorporated fuzzy logic to reduce subjective assessments, using linguistic scales to assign fuzzy scores to risk factors [15]. However, these approaches may overlook specific vulnerabilities, such as those posed by hazardous chemicals, in their final risk assessments. Later, Suzon et al. presented a risk evaluation technique for explosive and flammable chemicals using fuzzy inference system [16]. The authors only used explosive and flammable chemicals, excluding toxic chemicals, without a proper precautionary system. Chen et al. [17] prompted a quantitative risk evaluation model to assess the risk of transportation accidents involving multi-vehicles. The authors used a dynamic Bayesian network to predict the leakage and explosion of hazardous chemicals. For various hazardous compounds, the model employed event trees to list possible situations and estimated the risk of domino accidents produced by each scenario. However, the authors did not consider the risk of these chemicals for storage or processing. Basheer et al. [18] presented the essence, uses, restrictions, and future directions of some effective approaches and techniques such as fault tree analysis (FTA), event tree analysis (ETA), hazard and operability (HAZOP) studies, Bayesian networks, quantitative risk analysis (QRA) etc.

In the realm of risk assessment, fuzzy logic has facilitated the development of methodologies to assess risk criticality and prioritize actions, as demonstrated in studies involving student performance evaluation [19], [20] and dynamic risk modeling, such as fire and lightning risk assessments. Li et al. [21] created a risk assessment model that employs fuzzy logic and takes human aspects into account. They created the risk assessment interface using the LabVIEW application. In order to reduce the hazards associated with workplace safety, Pinto et al. [22] developed a fuzzy QRAM model that takes chemistry, engineering, biomechanical data, and physical laws into consideration. A fuzzy mathematics-based method for evaluating the risk of lightning strikes that takes vulnerability, exposure, and hazard into consideration was given by Yu et al. [23]. A dynamic risk assessment technique was presented by Li et al. [24] to identify stored process dangers and lower the likelihood of accidents. This model assesses the dynamic risk of electrostatic discharge-related warehouse fires using machine learning algorithms and historical data. A model for forecasting the environmental danger of chemical combinations was proposed by Backhaus and colleagues [25]. At the moment, the potential effects of mixes are ignored when assessing the environmental dangers connected to chemicals, which can result in an underestimation of those risks. These applications highlight fuzzy logic's adaptability in managing uncertain data and supporting nuanced decision-making processes.

Gul and Guneri in [26] developed a fuzzy multi-criteria risk evaluation for an aluminum plate production factory using “the decision matrix technique”. They used the fuzzy analytic hierarchy process (FAHP) approach to rate the likelihood and severity of two risk variables associated with the risks. Then, using a fuzzy Technique for Order Preference by Similarity to the Ideal Solution (TOPSIS), 23 different hazard groups were ranked. The authors did not consider their research other than the aluminum industries. To assess hazards in a Turkish hospital, Gul et al. [27] proposed a two-stage fuzzy multi-criteria approach that included FAHP and fuzzy VlseKriterijumska Optimizacija I Kompromisno Resenje (VIKOR) approaches. They used the FAHP methodology to score five risk indicators, and the fuzzy VIKOR method was used to rank the different sorts of hazards according to hospital sectors. Ozdemir et al. [28] proposed a novel risk evaluation methodology for a university chemical laboratory that included the 5S approach, failure mode and effects analysis (FMEA), interval type-two fuzzy sets (IT2FSs), analytic hierarchy process (AHP), and VIKOR. In the assessment step of the FMEA's occurrence, severity, and detection parameters, they integrated AHP into IT2FSs. A fuzzy-logic-based technique for automating ergonomic risk assessment in building projects was presented by Govindan and Li [29]. The authors presented an approach for assessing the impact and likelihood of risks by combining a risk matrix with fuzzy logic. The goal of this model is to offer a useful and simple method for assessing project risk. Furthermore, Łapczyńska and Burduk [30] employed the Mamdani technique in their study, proposing a fuzzy inference system to assess the risks associated with production process machinery.

Fuzzy logic is applied to risk assessment in assembly and forming industrial processes in the article in [31]. By using the fuzzy FMEA method, risk parameters might be evaluated based on the opinions of experts. Because of this, a system that is more adaptable and resilient to human error-related mistakes has been developed, facilitating the use of language variables in risk assessment. The purpose of the study [32] is to suggest a comprehensive method for evaluating security and safety concerns in chemical process industries. In order to do this, a taxonomy of safety and security risk factors has been devised, which consists of twenty contributing elements and four dimensions (i.e., occurrence likelihood, severity, vulnerability, and securing). The fuzzy analytical hierarchy process and statistical analysis were used in conjunction with the Delphi technique, which involved subject matter experts (N = 25) to evaluate the taxonomy's validity and reliability. Solukloei et al. [33] presented a hybrid methodology that integrates an Ant Colony System (ACS), fuzzy set theory, and fuzzy-HAZOP technique to assess the risk of hazardous discharge, fire, and explosion in process industries. Fuzzy analytical hierarchy processes (AHP) were used by Jabbari et al. [34] to evaluate the hazards associated with toxic gas discharge, explosion, and fire. Safety managers obtained useful information from this model to aid in their decision-making. Fuzzy Bayesian networks (FBN) were introduced by Zhang et al. [35] to evaluate the safety of heavy oil pipelines. FBN was used to alleviate the data shortage and offer a reliable model for risk assessment. Using a fuzzy technique, Pahlevan et al. [36] examined the effects of an offshore pipeline breakdown. Offshore pipeline risk assessment was made easier by using a methodical methodology. The study in [37] showed the usefulness of hypothetical data in pipeline risk assessment by evaluating the risks related to interstate pipelines. However, it's crucial to remember that because the modeling was predicated on hypothetical data, its applicability in actual situations might be restricted.

In conclusion, while fuzzy logic enhances the robustness of risk assessment methodologies by accommodating uncertainties in data, further research is needed to refine its application across diverse risk scenarios and to address specific vulnerabilities comprehensively.

## 3 Methodology

### 3.1 Overall system architecture

**Figure 1:**
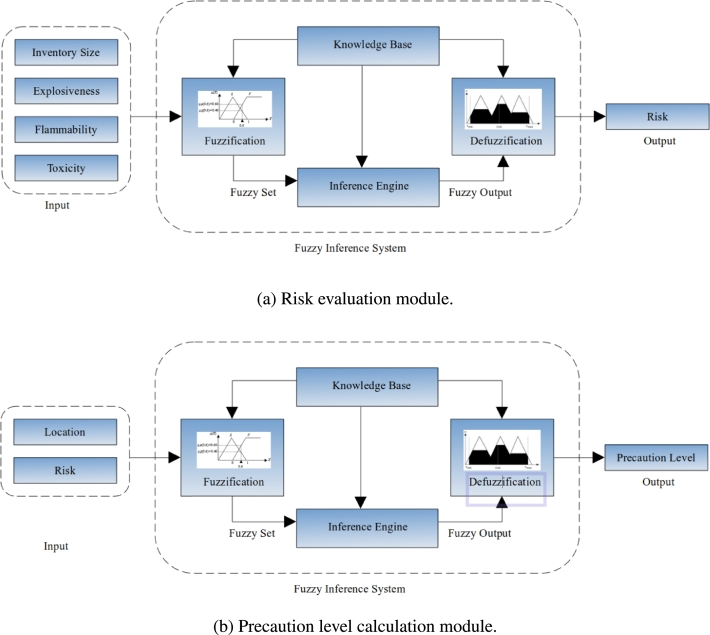
The proposed system architecture.

The proposed system is comprised of two modules, as represented in Fig. 1. One is in Fig. 1a, for the risk evaluation module, where inventory sizes, explosiveness, flammability, and toxicity of materials are considered as input variables for fuzzification and generate the risk for the industries. The second module is in Fig. 1b, responsible for generating the precaution level to be taken by the industries to minimize the damage. This module considers the risk evaluated in the first module and the location of the industries as input variables for fuzzification. The fuzzified values in both modules are sent to the knowledge base, where the fuzzy rules evaluation and aggregation occur. Finally, the defuzzification step returns a crisp value indicating the output (risk and precaution level) of the corresponding module.

### 3.2 Mathematical background and example

As the system considers two modules, their working mechanisms will be described here. Each module uses the Mumdani-type fuzzy inference system, and their working procedures are the same except for the number of input variables. Hence, both the risk evaluation and precaution level calculation modules conduct their operations through the following steps.

#### 3.2.1 Fuzzification of input variables

Fuzzification is the process of converting a crisp (non-fuzzy) input into a fuzzy input in fuzzy logic systems. It is a crucial step in the implementation of fuzzy systems, as it provides the necessary information for the inference engine to make a decision. The risk evaluation module has 4 input variables named inventory size, explosiveness, flammability, and toxicity, and one output variable named risk. The precaution level calculation module has two input variables named location and risk and one output variable, precaution level. The input variables of both modules, linguistic values, and their representing symbols are described in Table 1, and the output variables are described in Table 2.

**Table 1:** The input variables of the system and their linguistic values.

Input Variables	Linguistic Values
Inventory Size	Small (1), Medium (2), Large (3)
Explosiveness	Low (1), Medium (2), High (3)
Flammability	Low (1), Medium (2), High (3)
Toxicity	Low (1), Medium (2), High (3)
Location	Rural (1), Urban (2), High Density (3)
Risk	Very Low (1), Low (2), Medium (3), High (4), Very High (5)

**Table 2:** The output variables of the system and their linguistic values.

Output Variables	Linguistic Values
Risk	Extremely Low (1), Very Low (2), Low (3), Medium (4), High (5), Very High (6), Extremely High (7)
Precaution Level	Very Low (1), Low (2), Medium (3), High (4), Very High (5)

**Figure 2:**
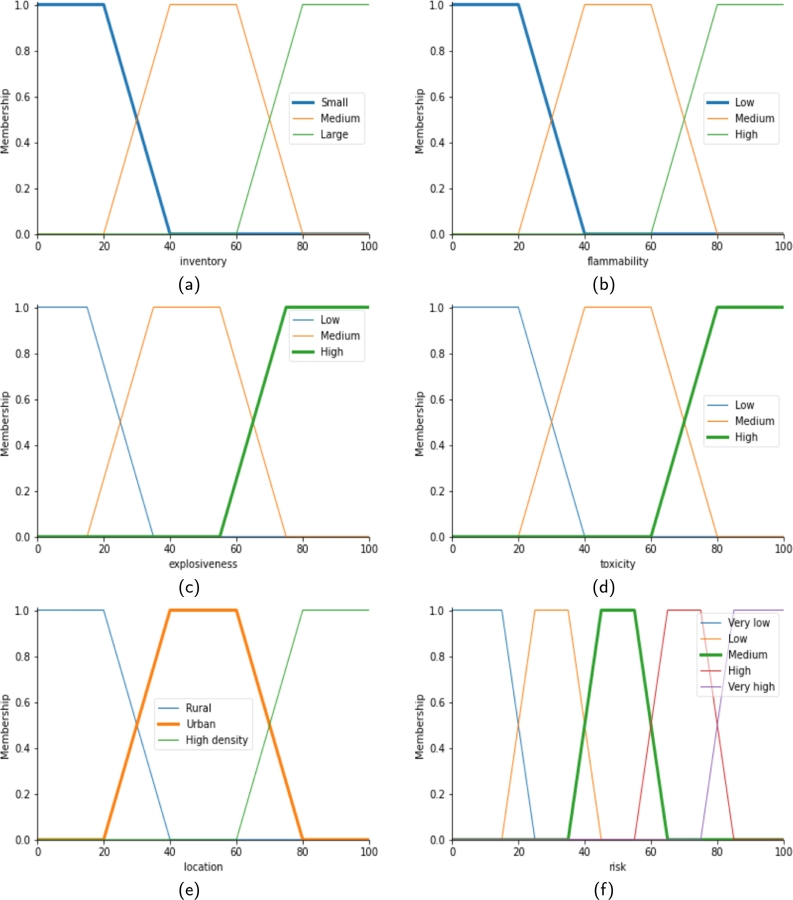
The membership function for several input variables considered for the system: (a) Inventory Size, (b) Flammability, (c) Explosiveness, (d) Toxicity, (e) Location, and (f) Risk.

In a typical fuzzy system, the inputs are first converted into fuzzy sets using a membership function. This function assigns a degree of membership between 0 and 1 to each input, indicating the degree to which it belongs to a particular fuzzy set. The degree of membership provides the fuzzy system with a more nuanced representation of the inputs, allowing for a more flexible and intuitive decision-making process. There are several ways to perform fuzzification, including the use of triangular, trapezoidal, and Gaussian membership functions. A trapezoidal membership function, which would be used in this study, is represented as (1) and visualized for each of the input variables in Fig. 2. In this membership function, *x* represents the input value in the range between (1-100), and a, b, c, and d represent the *x*-axis values of four corners of the trapezoid for a linguistic value of an input variable. For example, considering the input variable of inventory size shown in Fig. 2a, there are three linguistic values: Small, Medium, and Large. Each of the values is presented by the trapezoidal membership function. The values of (a, b, c, d) are (0, 0, 20, 40), (20, 40, 60, 80), and (60, 80, 100, 100) for the linguistic values, Small, Medium, and Large respectively. Similarly, these values may change for other input variables such as flammability, explosiveness, toxicity, location, and risk drawn in Fig. 2.

(1) $$\mu (x,a,b,c,d)=\left\{ \begin{array}{cc} \frac{x-a}{b-a}, & a\leq x\leq b.\\ 1, & b<x<c.\\ \frac{d-x}{d-c}, & c\leq x\leq d.\\ 0, & \text{otherwise}. \end{array}\right.$$

The trapezoidal function is the most appropriate method for representing the input variables in this study because it allows for full membership across a range of values rather than just at a single point as with the triangular function. This makes the trapezoidal function more suitable for the data points being used. Some other reasons behind using this function are its simplicity of construction and effectiveness in computational cost. In addition, this function generates a reconstruction error of zero at a 50% overlapping level [38], [39], [40], [41].

From Fig. 1, it is shown that the risk evaluation module has four input variables: Inventory Size, Flammability, Explosiveness, and Toxicity. Each variable considers three linguistic values or three levels (*n=3*) of input for the fuzzy sets. The precaution level calculation module has two input variables: Location, and Risk. These two consider three and five linguistic values or three and five levels (*n=3, 5*) of input for the fuzzy sets, respectively.

The quantification method or the value index system of these input variables can be discussed in several measurement units. The inventory size can be measured in cubic meters. Suppose the maximum allowable inventory size is *Y* cubic meters for a certain square foot of industrial area, and the available inventory size of the industry is *Z* cubic meters. Then the relative inventory size (0-100) for the system is calculated as equation (2).

(2) $$Inventory\;Size=\frac{Z}{Y}\times 100$$

A material's explosiveness and flammability are commonly quantified using the terms lower explosive/flammable limit and upper explosive/flammable limit. These limits, defined as percentages (0-100) by volume, specify the level range at which an explosive/flammable gas or vapor may explode/flame while mixing with air using the explosimeter (combustible gas detector) [42], [43]. There are several units for quantifying the toxicity of chemical materials. In this research, the percent concentration in the air (0-100) is considered to measure the toxicity of chemical compounds using the instrument gas chromatograph [44].

The input location of an industry can be given to the system as the density of the population (*P*) per square kilometer ($people/km^{2}$). Then the system converts it into a relative location (0-100) based on the density using the min-max normalization [45]. If the range of density of the population varies from *M* to *D*, then the input value of the location is calculated as (3).

(3) $$Location=\frac{P-M}{D-M}\times (100-0)+0=\frac{P-M}{D-M}\times 100$$

Here, *M* and *D* represent the lowest and highest population densities that have been considered for the industrial area. *P* is the current density of the population where the industry is located. For example, this research considers an industry that is located in an area where the population density varies from 500 to 1500 $people/km^{2}$. If the current density in that area is 1260 $people/km^{2}$, then the relative location value is 76, determined by equation (3). Finally, the unit-less risk and precaution level represent the likelihood of a risk occurring, and the precautionary level should be taken into consideration to minimize the damages. These values range between 0 and 100.

Based on the input variables and the fuzzy domain expert, the system generates the matrix IS, E, F, T, L, and R of dimension *1* × *n*. These matrices represent the degree of membership of each variable in the fuzzy domain and are represented as (4), (5), (6), (7), (8), and (9).

(4) $$IS=\left[\begin{array}{c} is_{i} \end{array}\right],1\times n.$$

(5) $$E=\left[\begin{array}{c} e_{i} \end{array}\right],1\times n.$$

(6) $$F=\left[\begin{array}{c} f_{i} \end{array}\right],1\times n.$$

(7) $$T=\left[\begin{array}{c} t_{i} \end{array}\right],1\times n.$$

(8) $$L=\left[\begin{array}{c} l_{i} \end{array}\right],1\times n.$$

(9) $$R=\left[\begin{array}{c} r_{i} \end{array}\right],1\times n.$$

where $is_{i}$, $e_{i}$, $f_{i}$, $t_{i}$, $l_{i}$, and $r_{i}$ denote the degree of membership in the level *i* for the corresponding input variable inventory size, explosiveness, flammability, toxicity, location, and risk, respectively.

The input level i=1 denotes the linguistic value “Small” or “Low” or “Rural” or “Very Low”, i=2 denotes “Medium”, or “Urban” or “Low”, i=3 denotes “Large” or “High” or “High Density”, i=4 denotes “High”, and i=5 denotes “Very High” for the several input variables listed in Table 1.

#### 3.2.2 Fuzzy rules generation and aggregation

The fuzzy rule base is a critical component of a fuzzy inference system, as it defines the relationships between the inputs and the outputs of the system. The fuzzy rule base consists of a set of fuzzy IF-THEN rules that specify the conditions under which a particular output should be generated. Each rule is defined in terms of a set of fuzzy antecedents (the inputs) and a set of fuzzy consequents (the outputs). The antecedents and consequents are represented as fuzzy sets, and the degree of membership of an input to a particular fuzzy set is used to evaluate the degree of support for a particular rule. The design of the fuzzy rule base is crucial to the performance of a fuzzy system, as it determines the accuracy and reliability of the system's outputs. So, Table 3 lists the fuzzy rules for evaluating the risk of the system, and Table 4 lists the rules for calculating the precaution level. The first rule from both tables can be written as ‘IF *Inventory Size* is Small and *Explosiveness* is Low and *Flammability* is Low and *Toxicity* is Low THEN *Risk* is Extremely Low’ and ‘IF *Location* is Rural and *Risk* is Very Low THEN *Precaution Level* is Very Low’. The other rules can be written in the same way.

**Table 3:** The fuzzy inference rules for the risk evaluation.

SI	Inventory Size	Explosiveness	Flammability	Toxicity	Risk
1	Small (1)	Low (1)	Low (1)	Low (1)	Extremely Low (1)
2	Small (1)	Low (1)	Low (1)	Medium (2)	Extremely Low (1)
3	Small (1)	Low (1)	Low (1)	High (3)	Very Low (2)
4	Small (1)	Low (1)	Medium (2)	Low (1)	Very Low (2)
5	Small (1)	Low (1)	Medium (2)	Medium (2)	Very Low (2)
6	Small (1)	Low (1)	Medium (2)	High (3)	Low (3)
7	Small (1)	Low (1)	High (3)	Low (1)	Low (3)
8	Small (1)	Low (1)	High (3)	Medium (2)	Low (3)
9	Small (1)	Low (1)	High (3)	High (3)	Low (3)
10	Small (1)	Medium (2)	Low (1)	Low (1)	Very Low (2)
11	Small (1)	Medium (2)	Low (1)	Medium (2)	Very Low (2)
12	Small (1)	Medium (2)	Low (1)	High (3)	Low (3)
13	Small (1)	Medium (2)	Medium (2)	Low (1)	Low (3)
14	Small (1)	Medium (2)	Medium (2)	Medium (2)	Low (3)
15	Small (1)	Medium (2)	Medium (2)	High (3)	Medium (4)
16	Small (1)	Medium (2)	High (3)	Low (1)	Low (3)

...	...	...	...	...	...

42	Medium (2)	Medium (2)	Medium (2)	High (3)	High (5)
43	Medium (2)	Medium (2)	High (3)	Low (1)	High (5)
44	Medium (2)	Medium (2)	High (3)	Medium (2)	High (5)
45	Medium (2)	Medium (2)	High (3)	High (3)	Very High (6)
46	Medium (2)	High (3)	Low (1)	Low (1)	High (5)
47	Medium (2)	High (3)	Low (1)	Medium (2)	High (5)
48	Medium (2)	High (3)	Low (1)	High (3)	High (5)
49	Medium (2)	High (3)	Medium (2)	Low (1)	Very High (6)
50	Medium (2)	High (3)	Medium (2)	Medium (2)	Very High (6)
51	Medium (2)	High (3)	Medium (2)	High (3)	Extremely High (7)
52	Medium (2)	High (3)	High (3)	Low (1)	Very High (6)
53	Medium (2)	High (3)	High (3)	Medium (2)	Very High (6)
54	Medium (2)	High (3)	High (3)	High (3)	Extremely High (7)
55	Large (3)	Low (1)	Low (1)	Low (1)	Low (2)

...	...	...	...	...	...

63	Large (3)	Low (1)	High (3)	High (3)	High (5)
64	Large (3)	Medium (2)	Low (1)	Low (1)	High (5)
65	Large (3)	Medium (2)	Low (1)	Medium (2)	High (5)
66	Large (3)	Medium (2)	Low (1)	High (3)	Very High (6)
67	Large (3)	Medium (2)	Medium (2)	Low (1)	High (5)
68	Large (3)	Medium (2)	Medium (2)	Medium (2)	Very High (6)
69	Large (3)	Medium (2)	Medium (2)	High (3)	Very High (6)
70	Large (3)	Medium (2)	High (3)	Low (1)	Very High (6)
71	Large (3)	Medium (2)	High (3)	Medium (2)	Very High (6)
72	Large (3)	Medium (2)	High (3)	High (3)	Extremely High (7)
73	Large (3)	High (3)	Low (1)	Low (1)	Very High (6)
74	Large (3)	High (3)	Low (1)	Medium (2)	Very High (6)
75	Large (3)	High (3)	Low (1)	High (3)	Extremely High (7)
76	Large (3)	High (3)	Medium (2)	Low (1)	Very High (6)
77	Large (3)	High (3)	Medium (2)	Medium (2)	Extremely High (7)
78	Large (3)	High (3)	Medium (2)	High (3)	Extremely High (7)
79	Large (3)	High (3)	High (3)	Low (1)	Extremely High (7)
80	Large (3)	High (3)	High (3)	Medium (2)	Extremely High (7)
81	Large (3)	High (3)	High (3)	High (3)	Extremely High (7)

**Table 4:** The fuzzy inference rules for the precaution level calculation.

SI	Location	Risk	Precaution Level
1	Rural (1)	Very Low(1)	Very Low (1)
2	Rural (1)	Low (2)	Very Low (1)
3	Rural (1)	Medium (3)	Low (2)
4	Rural (1)	High (4)	Medium (3)
5	Rural (1)	Very High(5)	High (4)
6	Urban (2)	Very Low(1)	Low (2)
7	Urban (2)	Low (2)	Medium (3)
8	Urban (2)	Medium (3)	Medium (3)
9	Urban (2)	High (4)	High (4)
10	Urban (2)	Very High(5)	Very High (5)
11	High density(3)	Very Low(1)	Medium (3)
12	High density(3)	Low (2)	High (4)
13	High density(3)	Medium (3)	High (4)
14	High density(3)	High (4)	Very High (5)
15	High density(3)	Very High(5)	Very High (5)

Now, for aggregation of fuzzy rules, a matrix $AG_{x}$ of dimension *1* × *n* is generated as (10),

(10) $$AG_{x}=\left[\begin{array}{c} ag_{i} \end{array}\right],1\times n.$$

where $ag_{i}$ is the aggregated value for the variable *x* on its linguistic value *i*. The value of $ag_{i}$ can be calculated by (11) and (12) for the risk evaluation module and precaution level calculation level, respectively, as:

(11) $$ag_{i}=max_{\left\{(k,p,m,q)|Rule(k,p,m,q)=i\right\}}\{min(is_{k},e_{p},f_{m},t_{q})\}$$

where *(k, p, m, q)|Rule(k, p, m, q) = i* indicates those rules for which the output variable Risk is *i* if the input *is* is *k*, *e* is *p*, *f* is *m*, and *t* is *q* from the Table 3.

(12) $$ag_{i}=max_{\left\{(k,p)|Rule(k,p)=i\right\}}\{min(l_{k},r_{p})\}$$

where *(k, p)|Rule(k, p) = i* indicates those rules for which the output variable Precaution Level is *i* if the input *l* is *k*, and *r* is *p* from the Table 4.

#### 3.2.3 Defuzzification

Defuzzification is a process used in Fuzzy Inference Systems (FIS) to convert the fuzzy output of a FIS into a crisp value. The objective of defuzzification is to convert the vague and imprecise output of an FIS into a single numerical value that can be easily understood and interpreted. It is the final step in the inference process of an FIS and provides the system with the ability to make a definite decision based on the processed information.

Defuzzification is achieved by using various methods, including the centroid method, the bisector method, and the mean of the maximum method. The centroid method, also known as the center of gravity (CoG) method, involves finding the center of the fuzzy output set by dividing the total area under the membership function by the area of the set.

Each defuzzification method has its strengths and weaknesses, and the choice of method depends on the specific requirements of the problem being solved. For instance, the centroid method is regarded as the most precise approach, but it is also the most computationally intensive method. It is important to note that defuzzification is not a one-size-fits-all solution and the choice of method should be made carefully, taking into account the specific requirements of the problem being solved.

In some cases, a combination of defuzzification methods may be necessary to obtain the desired results. The current study employs the center of gravity defuzzifier for defuzzification, as it calculates the mean value of the parameters [46]. It is mathematically represented as (13).

(13) $$CoG=\frac{\int_{x_{min}}^{x_{max}}\mu (x).xdx}{\int_{x_{min}}^{x_{max}}\mu (x)dx}$$

Here, *x* is a value in the range of the output variable. For every value *x* in the fuzzy output set, μ(x) is the membership function value (degree of membership). $x_{min}$ is the lowest value that can exist in the output variable's domain and $x_{max}$ represents the output domain's maximum value.

#### 3.2.4 An example of risk evaluation and precaution level calculation

Let's use an example to illustrate further. The system has a total of six input variables from two modules. For the first module, consider that the values of the four variables are Inventory Size = 87, Explosiveness = 56, Flammability = 45, and Toxicity = 67. In the fuzzification step, these values have been passed to the membership function, as in (1) and Fig. 2, then get the IS, E, F, and T matrices as follows:

$$IS=\left[\begin{array}{c} is_{i} \end{array}\right]=\left[\begin{array}{ccc} 0 & 0 & 1 \end{array}\right],1\times 3.$$

here, $is_{3}=1$ represents the degree of membership value for the given inventory size in the level of Large (i=3).

$$E=\left[\begin{array}{c} e_{i} \end{array}\right]=\left[\begin{array}{ccc} 0 & 0.95 & 0.05 \end{array}\right],1\times 3.$$

here, $e_{2}=0.95$ represents the degree of membership value for the given explosiveness in the level of Medium (i=2).

$$F=\left[\begin{array}{c} f_{i} \end{array}\right]=\left[\begin{array}{ccc} 0 & 1 & 0 \end{array}\right],1\times 3.$$

here, $f_{2}=1$ represents the degree of membership value for the given flammability in the level of Medium (i=2).

$$T=\left[\begin{array}{c} t_{i} \end{array}\right]=\left[\begin{array}{ccc} 0 & 0.65 & 0.35 \end{array}\right],1\times 3.$$

here, $t_{3}=0.35$ represents the degree of membership value for the given toxicity in the level of High (i=3).

Now, to get the $AG_{x}$ matrix using (11) considering the output variable x=Risk and i=1 which represent the value of the output variable Risk is Extremely Low. Then,

$$ag_{1}=max_{\left\{(k,p,m,q)|((1,1,1,1),(1,1,1,2))\right\}}\{min(is_{k},e_{p},f_{m},t_{q})\}=max\{min(is_{1},e_{1},f_{1},t_{1}),min(is_{1},e_{1},f_{1},t_{2})\}=max\{min(0,0,0,0),min(0,0,0,0.65)\}=max\{0,0\}=0$$

Similarly for i=2.

$$ag_{2}=max_{\left\{(k,p,m,q)|((1,1,1,3),(1,1,2,1),(1,1,2,2),(1,2,1,1),(1,2,1,2),(2,1,1,1),(2,1,1,2))\right\}}\{min(is_{k},e_{p},f_{m},t_{q})\}=max\{min(0,0,0,0.35),min(0,0,1,0),min(0,0,1,0.65),min(0,0.95,0,0),min(0,0.95,0,0.65),min(0,0,0,0),min(0,0,0,0.65)\}=0$$

In the same way, $ag_{3}=0,ag_{4}=0,ag_{5}=0,ag_{6}=0.65,ag_{7}=0.05$. So, the $AG_{Risk}$ matrix is as follows.

$$AG_{Risk}=\left[\begin{array}{c} ag_{i} \end{array}\right]=\left[\begin{array}{ccccccc} 0 & 0 & 0 & 0 & 0 & 0.65 & 0.05 \end{array}\right],1\times 7.$$

**Figure 3:**
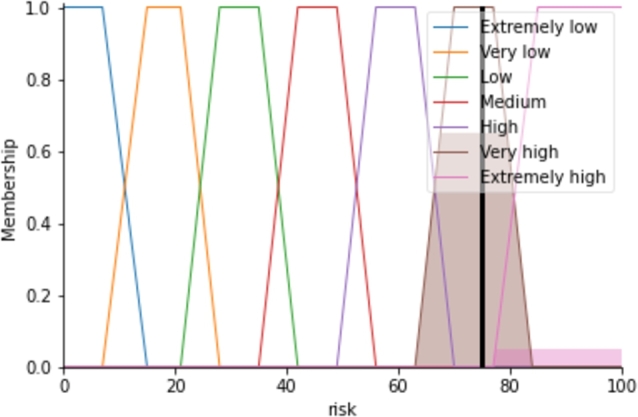
The final risk evaluation for the given input of the system.

The defuzzification uses the matrix $AG_{Risk}$ and the equation (13) to evaluate the final Risk value that is 74.81 for the system represented as in Fig. 3. For evaluating the final risk value, (13) integrates the colored areas of the ‘Very high’ and ‘Extremely high’ in Fig. 3 where the *y*-axis values of them are 0.65 and 0.05 respectively used from the $AG_{Risk}$ matrix. The entire system is implemented using the Python SciKit-Fuzzy package [47].

Likewise, for the second module, considering the values of the two input variables, Location = 78 and Risk = 74.81. In the fuzzification step, these values have been passed to the membership function, as in (1) and Fig. 2, then get the L, and R matrices as follows:

$$L=\left[\begin{array}{c} l_{i} \end{array}\right]=\left[\begin{array}{ccc} 0 & 0.10 & 0.90 \end{array}\right],1\times 3.$$

Here, $l_{3}=0.90$ represents the degree of membership value for the given location in the level of High density (i=3).

$$R=\left[\begin{array}{c} r_{i} \end{array}\right]=\left[\begin{array}{ccccc} 0 & 0 & 0 & 1 & 0 \end{array}\right],1\times 3.$$

Here, $r_{4}=1$ represents the degree of membership value for the given risk in the level of High (i=4).

Now, to get the $AG_{x}$ matrix using (12) considering the output variable x=PrecautionLevel and i=1 which represents the value of the output variable Precaution Level is Very Low. Then,

$$ag_{1}=max_{\left\{(k,p)|((1,1),(1,2))\right\}}\{min(l_{k},r_{p})\}=max\{min(l_{1},r_{1}),min(l_{1},r_{2})\}=max\{min(0,0),min(0,0)\}=max\{0,0\}=0$$

Similarly, $ag_{2}=0,ag_{3}=0,ag_{4}=0.10,ag_{5}=0.90$. So, the $AG_{Precaution\;Level}$ matrix is as follows.

$$AG_{Precaution\;Level}=\left[\begin{array}{c} ag_{i} \end{array}\right]=\left[\begin{array}{ccccc} 0 & 0 & 0 & 0.10 & 0.90 \end{array}\right],1\times 5.$$

**Figure 4:**
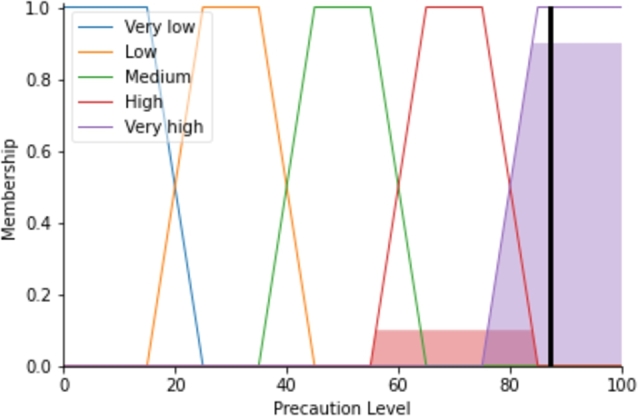
The precaution level calculation for the given input of the system.

Following that, the defuzzification uses the equation (13) and the matrix $AG_{Precuation\;Level}$ to evaluate the precaution level that is 87.23 for the system represented as in Fig. 4. For calculating the precaution level, (13) integrates the colored areas of the ‘High’ and ‘Very high’ in Fig. 4 where the *y*-axis values of them are 0.10 and 0.90, respectively, used from the $AG_{Precuation\;Level}$ matrix.

## 4 Experimental analysis and discussion

In this study, a risk assessment of hazardous chemicals specifically explosive, flammable, and toxic substances, is conducted to evaluate the potential dangers associated with these chemicals. Various combinations of crisp input values are utilized to calculate the risk posed by these harmful substances when input values are synthetically generated. The results, displayed in Table 5, indicate that risk increases as the values for explosiveness, flammability, and toxicity rise. In the risk calculation, explosiveness is assigned the highest priority, while toxicity is given the lowest priority. The results indicate that an increase in explosiveness results in a significantly greater increase in risk compared to increases in toxicity or flammability. Inventory size, which refers to the quantity of these chemicals along with other substances, also plays a crucial role. The data show that as the inventory size increases, the risk correspondingly rises. This is because a larger amount of hazardous chemicals can cause more extensive damage.

**Table 5:** Evaluated risk based on the inputs.

Inventory Size	Explosiveness	Flammability	Toxicity	Risk
45	23	34	34	32.65
45	45	34	34	41.03
45	65	34	34	54.87
39	30	34	44	35.83
39	30	55	44	40.47
39	30	75	44	49.37
50	45	34	22	41.03
50	45	34	44	45.5
50	45	34	65	49.44
25	45	34	34	30.98
76	23	56	56	48.99
45	56	56	34	47.85
14	18	34	45	17.78
34	23	78	33	44.44
54	56	23	67	52.17
56	65	34	22	54.26
80	23	23	34	46.83
70	67	34	45	70.38
56	77	22	10	61.14
89	56	12	67	65.97
54	79	23	23	61.89
45	45	76	23	56.38
90	78	45	45	90.35
56	54	34	68	51.25
78	56	23	36	59.19
87	23	23	56	46.83
23	33	23	15	22.83
67	55	35	27	52.57
76	54	56	45	55.38
56	34	23	45	43.09
87	56	45	67	74.80

The higher the risk value, the greater the potential for damage to people, property, and the environment in the event of an accident or terrorist activity. Previous studies primarily focused on the presence of hazardous chemicals. However, this study also considers the quantity of these chemicals and their specific dangerous properties, such as explosiveness, flammability, and toxicity. This comprehensive approach provides a clearer understanding of the potential damage these chemicals can cause. The findings indicate that risk increases significantly with the amount of highly explosive or flammable chemicals.

The location of a chemical industry, which is calculated in terms of the density of the population using the method described in equation (3), significantly influences the level of precautions necessary to prevent accidents or terror attacks. In densely populated areas, the consequences of an incident can lead to higher casualties, extensive property damage, and greater environmental harm compared to rural areas. As indicated in Table 6, precaution levels are calculated based on both location and risk. Precautionary measures intensify considerably as the setting shifts from rural to highly populated areas. Additionally, higher risk levels necessitate elevated precautionary measures.

**Table 6:** Evaluated precaution level based on the inputs.

Population Density (P) ($People/km^{2}$)	Location using Eq. (3)	Risk	Precaution Level
1260	76	32.65	65.55
750	25	41.03	29.01
950	45	54.87	50.00
1170	67	35.83	57.31
840	34	40.47	35.73
1040	54	49.37	50.00
840	34	41.03	36.31
1260	76	45.5	65.55
950	45	49.44	50.00
1170	67	30.98	57.30
850	34	48.99	43.61
1080	58	47.85	50.00
1200	70	17.78	46.66
950	45	44.44	50.00
850	35	52.17	44.56
1060	56	54.26	50.00
5050	55	46.83	50.00
1040	54	70.38	70.0
720	22	61.14	44.70
1060	56	65.97	70.0
890	39	61.89	61.75
1280	78	56.38	69.96
700	20	90.35	70.0
730	23	51.25	33.42
950	45	59.19	58.55
1060	56	46.83	50.00
840	34	22.83	37.49
1270	77	52.57	66.57
1280	78	55.38	68.32
1300	80	43.09	69.99
730	23	74.80	53.42

The adage “Prevention is better than cure” underscores the importance of implementing adequate security measures to avert unintended incidents or terror attacks. Precaution levels can be categorized into five distinct levels, with Table 7, outlining the necessary steps for each level to prevent accidents. By comparing the current security status with the guidelines in Table 7, adjustments can be made to security arrangements as needed. For instance, if the current precaution level is low but the calculated level is high, steps should be taken to enhance the precautionary measures. Conversely, if the current precaution level is high but the calculated level is low, precautions can be reduced to minimize costs while maintaining safety.

**Table 7:** Recommended security arrangements based on risk.

Precaution Score	Level of Precaution	Recommendations
<10	Very Low	Follow basic safety procedures for handling (storing, processing, and transporting) hazardous chemicals. Keep an up-to-date inventory of hazardous chemicals and review safety protocols regularly.

11 − 30	Low	Keep hazardous chemicals in a designated location with labeled containers. Implement additional security measures, such as locks or security cameras. Implement more stringent procedures for handling and disposing of hazardous chemicals. Regularly inspect and maintain equipment used for handling hazardous chemicals.

31 − 60	Medium	Implement additional security measures, such as security guards and perimeter fencing. Implement stringent procedures for handling and disposing of hazardous chemicals. Conduct frequent training and drills for employees on emergency response procedures. Regularly inspect and maintain equipment used for handling hazardous chemicals.

61 − 80	High	Deploy a large number of well-trained security guards and install robust fencing. Implement stricter guidelines for handling and disposing of hazardous chemicals. Conduct frequent training and drills for employees on emergency response procedures. Increase the frequency of security inspections. Use specialized containment and storage facilities.

>81	Very High	Implement extensive security measures, such as well-trained security guards and multi-layer perimeter fencing. Follow strict rules and regulations to handle hazardous chemicals. Conduct frequent training for employees on emergency response procedures. Regularly inspect and maintain equipment used for handling hazardous chemicals. Implement advanced measures to prevent accidental releases or spills, such as emergency shutdown systems or automated spill response systems. Have a dedicated and capable team in place to monitor and manage the security of hazardous chemicals on a regular basis.

## 5 Conclusion

The chemical industry manages a vast inventory of hazardous substances, increasing the potential for accidents and terrorist incidents. Recent occurrences underscore the significant security challenges posed by such events near chemical facilities. Therefore, robust risk assessment methods accompanied by security measures are crucial. This study focuses on evaluating risk by considering the types and explosiveness, flammability, toxicity, and the amount of chemicals involved. By integrating these factors, the study aims for more precise risk estimation that previous methods may have overlooked. Higher explosiveness or flammability increases the potential severity of accidents, influencing precautionary measures and security protocols. However, there are some limitations, particularly in collecting real-world data from the chemical factories in the described format, as the industries in a country like Bangladesh lack the ability to measure the risk parameters. Consequently, synthetic data was used to generate the results. Additionally, other risk factors, such as storage management systems and past incidents of terrorism, could have been considered in calculating the precaution level to suggest security levels more accurately.

Future research could enhance these assessments by incorporating additional parameters to refine further risk evaluations and precautionary strategies. Developing more robust methods for measuring the intensity of hazardous properties and other inputs would also be valuable. Future research could explore incorporating alternative prediction methods [48] and conduct model comparisons for improved analysis. Collecting real-world data on chemical reactions could further enhance the accuracy of future studies. In summary, the findings of the paper emphasize the significance of maintaining proper safety protocols and remaining vigilant to protect human lives and the environment. With further advancement, this work could serve as a foundation for more comprehensive safety measures across the chemical industry.

## Funding

This research did not receive any specific grant from funding agencies in the public, commercial, or not-for-profit sectors.

## CRediT authorship contribution statement

**Abdul Aziz:** Writing – review & editing, Writing – original draft, Validation, Supervision, Resources, Project administration, Methodology, Investigation, Conceptualization. **Md Masum Suzon:** Writing – review & editing, Writing – original draft, Visualization, Validation, Methodology, Formal analysis, Data curation, Conceptualization. **Rakib Hasan:** Writing – review & editing, Writing – original draft, Visualization, Methodology, Formal analysis, Data curation, Conceptualization.

## Declaration of Competing Interest

The authors declare that they have no known competing financial interests or personal relationships that could have appeared to influence the work reported in this paper.

## Data Availability

No dataset was used in this research article.
